# Supplementary material for: Product selectivity in plasmonic photocatalysis for carbon dioxide hydrogenation
Source: Nat Commun. 2017 Feb 23;8:14542. doi: 10.1038/ncomms14542 (PMC5348736; doi:10.1038/ncomms14542)
Supplement: Supplementary Information — Supplementary Figures, Supplementary Notes and Supplementary References [file ncomms14542-s1.pdf]

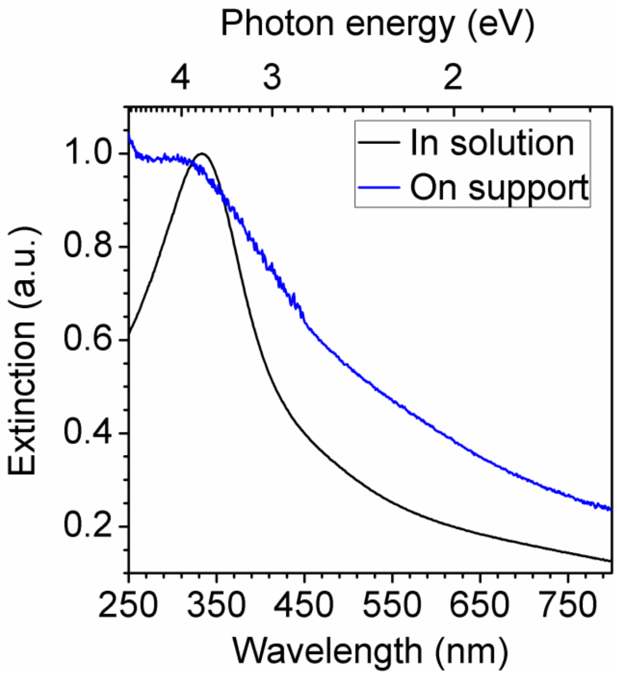

Supplementary Figure 1. Extinction spectra of rhodium nanocubes. UV-vis spectra of the Rh nanocubes in ethanol solution (black) and on a porous Al_2O_3 support (blue). The Rh nanocubes in ethanol solution show resonant wavelength of 334 nm, corresponding to the dipolar mode of localized surface plasmon resonance. The background from the solvent and quartz cuvette has been subtracted. This peak of plasmonic Rh nanocubes is blue-shifted and broadened when supported on an Al_2O_3 support due to the change of dielectric environment.

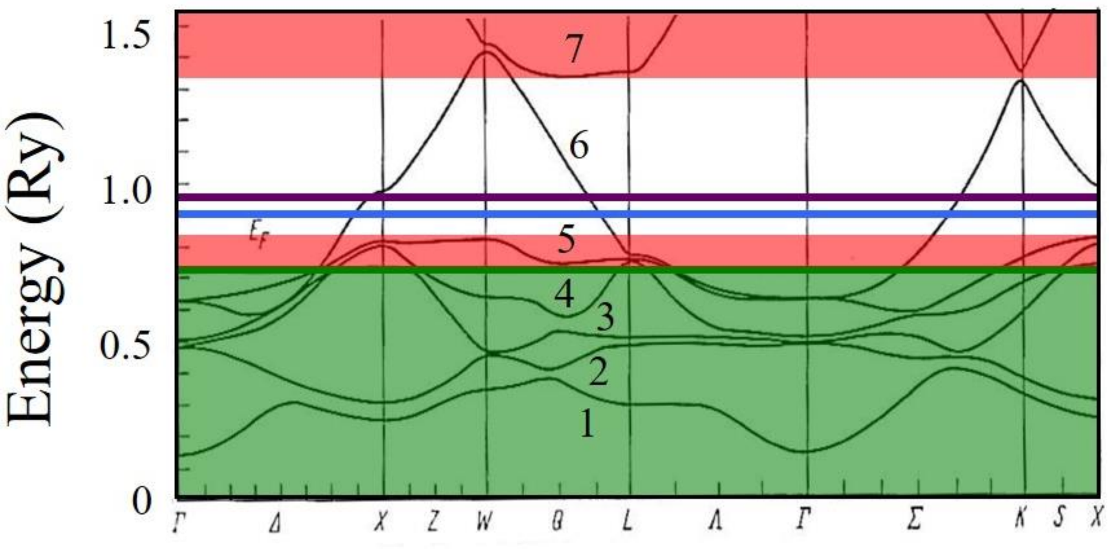

Supplementary Figure 2. Calculated band diagram of bulk rhodium. Bands 1-5 are partially filled to the Fermi level (green region, E_F is the solid green line). Interband transitions to the unfilled 4d band 5 (lower red region) dominate the optical behavior of Rh throughout infrared to 1.8 eV (700 nm). The quasi-quadratic 5sp band 6 allows nearly free-electron behaviors required for UV-Visible plasmonic resonances from 1.8 eV up to 8.6 eV (145 nm) where interband transitions to the 5sp band 7 begin to dominate (upper red region). The 3.4 and 2.7 eV photon energies (violet and blue lines, respectively) overlap the broad, near UV plasmonic resonance (3.71 eV, 334 nm) of our Rh photocatalyst powders and Rh cube suspensions, not the flat interband transitions (note that 1 Ry = 13.6 eV). Figure modified from ref. 1, copyright 1973 WILEY-VCH.

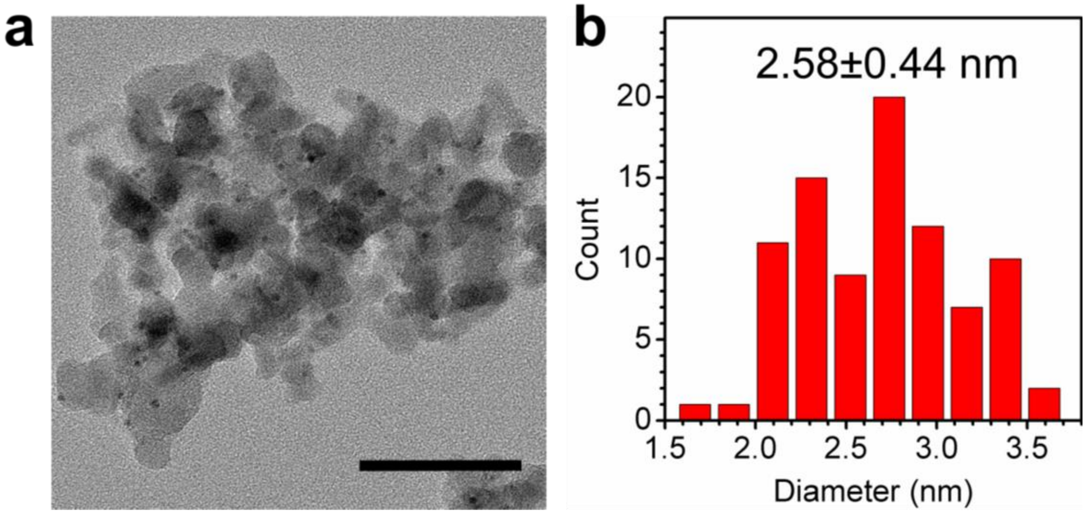

Supplementary Figure 3. Morphology of the Au/Al_2O_3 photocatalyst. (a) Transmission electron microscopic image of spherical Au nanoparticles supported on Al_2O_3 nanoparticles. Scale bar: 50 nm. (b) Size distribution of Au nanoparticles in the Au/Al_2O_3 photocatalyst. More than 80 nanoparticles were measured to obtain the size distribution.

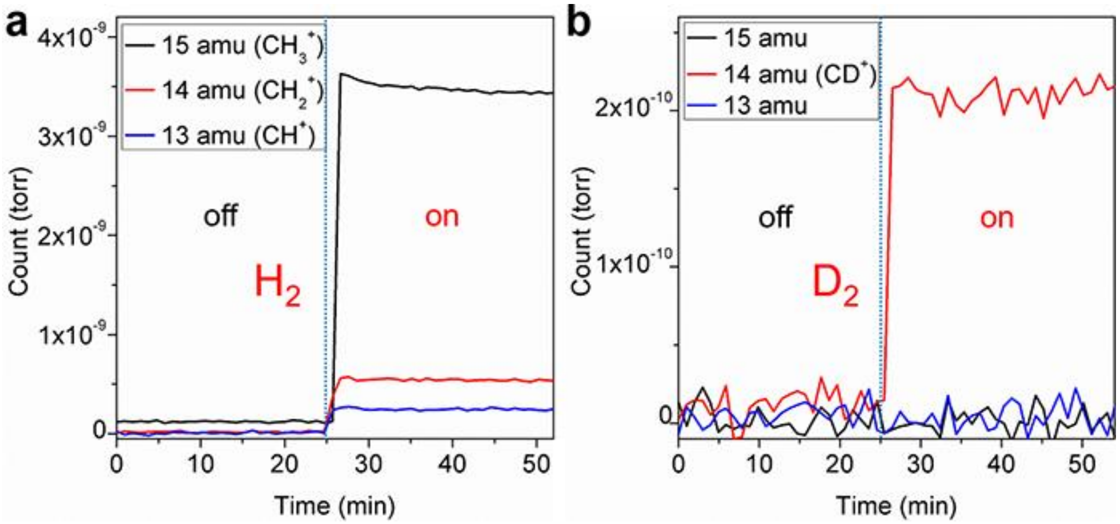

Supplementary Figure 4. Mass spectroscopy and isotopic labeling of methane reaction. (a) CO_2 hydrogenation reaction with H_2 on Rh/Al_2O_3 photocatalyst. Signals from 13 (blue), 14 (red) and 15 (black) atomic mass unit (amu) channels are detected by the mass spectrometer at 623 K, corresponding to CH^+, CH_2^+ and CH_3^+ fragments from CH_4. Under UV illumination at 3.0 W cm^{-2}, signals from these channels all increase, indicating the light-enhanced CH_4 production. (b) Isotopic labeling experiments with deuterium (D_2) on Rh/Al_2O_3 photocatalyst. Signal from only the 14 amu (red) channel is detected at 623 K and exhibits an enhancement upon UV illumination at 3.0 W cm^{-2}, corresponding to CD^+ fragments from CD_4. Signals from 13 (blue) and 15 amu (black) are not observed in the experiments. The intensity of CD^+ fragment is also comparable to that of the CH^+ fragment. These results confirm that the production of CH_4 is from the photocatalytic reaction on the Rh photocatalyst rather than from contaminants.

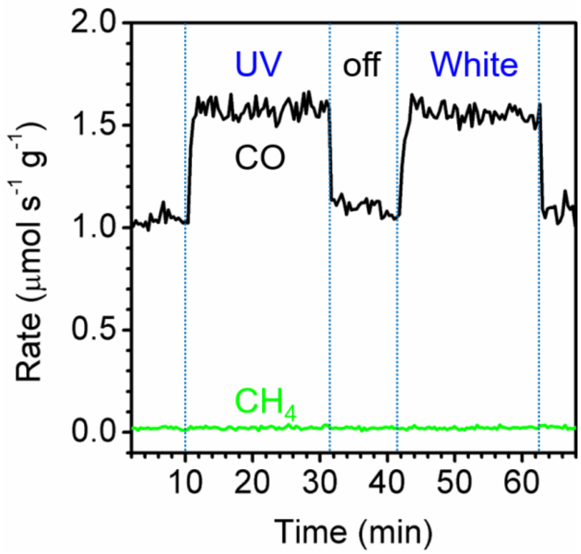

Supplementary Figure 5. Photocatalytic CO production on the Au/Al_2O_3 photocatalyst under UV and white light. The CO_2 hydrogenation reaction at 623 K under UV and white light of the same intensity (1.18 W cm^{-2}) exhibited practically the same reaction rates, and CO was the exclusive product on the Au photocatalyst at these conditions.

1. $CO_{2(g)} + 2\,* \rightleftharpoons CO* + O*$
2. $H_{2(g)} + 2\,* \rightleftharpoons 2\,H*$
3. $CO* \longrightarrow CO_{(g)} + *$ ·················· RDS for CO production
4. $CO* + H* \rightleftharpoons CHO* + *$
5. $CHO* + * \longrightarrow CH* + O*$ ······· RDS for CH_4 production
6. $O* + H* \rightleftharpoons OH* + *$
7. $OH* + H* \longrightarrow H_2O_{(g)} + 2\,*$
8. $CH* + H* \rightleftharpoons CH_2* + *$
9. $CH_2* + H* \rightleftharpoons CH_3* + *$
10. $CH_3* + H* \longrightarrow CH_{4(g)} + 2\,*$

Supplementary Figure 6. Elementary steps of CO_2 hydrogenation on the Rh/Al_2O_3 photocatalyst. The * denotes vacant sites or adsorbed intermediates on the Rh surface. The desorption of CO from the Rh surface is reported to be the rate-determining step (RDS) for CO production. The dissociation of CH-O into CH and O is reported to be the RDS for CH_4 production. The competition between these two steps dictates the product selectivity.

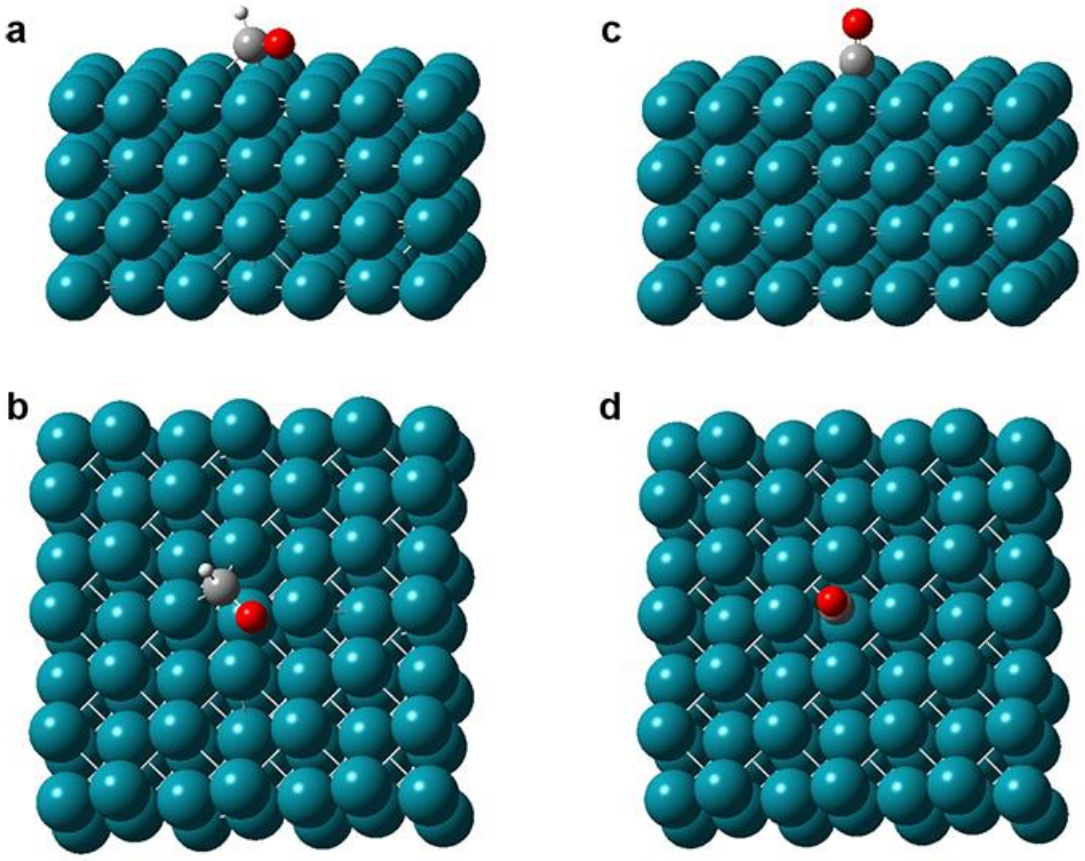

Supplementary Figure 7. Structures used in calculations of CHO and CO adsorbed on Rh surface. (a, b) side and top view of CHO adsorbed on the Rh(100) surface. (c, d) side and top view of CO adsorbed on the Rh(100) surface. The dark cyan, gray, red, and white spheres are Rh, C, O, and H atoms, respectively.

Supplementary Note 1. Plasmonic heating effects.

According to recent theoretical simulations[2-4], the temperature increase caused by plasmonic heating of the metal nanoparticles above its environment can be described by

$$\Delta T = \frac{\sigma_{\text{abs}} I}{4\pi R \beta \kappa} \quad (1)$$

where σ_{abs} is the absorption cross-section of metal nanoparticle, I is the light intensity, R is the radius of a spherical nanoparticle with the same volume as the modeled nanoparticle, β is a shape related factor, and κ is the thermal conductivity of surrounding environment. The simulated absorption cross-section of a 39 nm Rh nanocube immersed in ethanol is $\sigma_{\text{abs}} = 7.0 \times 10^{-15}$ m^2 at resonance (344 nm), as calculated by a method described previously[5]. The highest accessible light intensity in our experiments was I = 4.9 W cm^{-2}. The shape related factor is assumed to be $\beta = 1$ for a nanocube. The thermal conductivity of air and alumina are $\kappa \approx 0.03$ and 12 W m^{-1} K^{-1}, respectively. Based on these parameters, the upper limit of temperature increase above the ambient host temperature caused by plasmonic heating effects is $\Delta T = 0.05$ K. The small temperature increase from plasmonic heating further indicates that the light-enhanced reaction rates are mainly from the transfer of hot electrons, not from local heating effects.

Supplementary Note 2. Estimation of quantum yield.

The quantum yield of methane production on Rh photocatalysts is defined as

$$\varepsilon = \frac{R_{\text{methane}}}{R_{\text{photon}}} \times 100\% \quad (2)$$

The methane production rate is obtained by normalizing the 15 amu signal (CH_3^+) from the mass spectrometer to the 40 amu signal, corresponding to the internal standard, argon molecules. Since the flow rate of argon is known, the absolute methane production rate is obtained by normalizing to the argon flow rate. Taking the photocatalytic reaction at 623 K under the 460 nm blue light-emitting diode at 4.9 W cm^{-2} and with an H_2-rich condition, the 15 and 40 amu signals were 2.792×10^{-8} and 1.393×10^{-6} torr, respectively. Under dark condition, the 15 amu signal was 9.006×10^{-10} torr. The flow rate measured by the mass flow controller was at 25°C and 1 atm, corresponding to ~22400 mL/mol. The number of methane molecules formed by light in 1 s is

$$\frac{2.792 \times 10^{-8} - 9.006 \times 10^{-10}}{1.393 \times 10^{-6}} \times \frac{27.6\, mL/min \times 6.022 \times 10^{23}/mol}{22400\, mL/mol} \times \frac{1\, s}{60\, s/min}$$

$$= 2.40 \times 10^{17} \quad (3)$$

The photon energy of 460 nm light is 2.696 eV. The light intensity is 4.89 W cm^{-2}. The illuminated area of our catalyst bed is 0.2827 cm^2. The number of photons delivered to the catalyst in 1 s is

$$4.89\ W/cm^2 \times 0.2827\ cm^2 \times \frac{1\ s}{2.696\ eV\ \times 1.602 \times 10^{-19}\ J/eV} = 3.20 \times 10^{18} \quad (4)$$

The quantum yield is then

$$\varepsilon = \frac{2.40 \times 10^{17}}{3.20 \times 10^{18}} \times 100\% = 7.50\% \quad (5)$$

Supplementary References

1. Christensen, N. E. The Band Structure of Rhodium and Its Relation to Photoemission Experiments. *physica status solidi (b)* **55,** 117-127 (1973).

2. Baffou, Guillaume, Quidant, Romain & García de Abajo, F. Javier. Nanoscale Control of Optical Heating in Complex Plasmonic Systems. *ACS Nano* **4,** 709-716 (2010).

3. Christopher, Phillip, Xin, Hongliang & Linic, Suljo. Visible-light-enhanced catalytic oxidation reactions on plasmonic silver nanostructures. *Nat. Chem.* **3,** 467-472 (2011).

4. Govorov, Alexander O. & Richardson, Hugh H. Generating heat with metal nanoparticles. *Nano Today* **2,** 30-38 (2007).

5. Zhang, Xiao, Li, Pan, Barreda, Ángela, Gutiérrez, Yael, González, Francisco, Moreno, Fernando, Everitt, Henry O. & Liu, Jie. Size-tunable rhodium nanostructures for wavelength-tunable ultraviolet plasmonics. *Nanoscale Horiz.* **1,** 75-80 (2016).
